# Medical Journals and Practitioners

**Published:** 1888-02

**Authors:** L. Henderson

**Affiliations:** Salem, Oregon


### MEDICAL JOURNALS AND PRACTITIONERS.

L. HENDERSON, M. D., SALEM, OREGON.

We take the liberty of publishing extracts from this letter of Dr. Henderson, as it clearly tells the story of homœopathic journalism. The profession leave the work to the editors, and *then* blame them for issuing poor journals. Is it fair?—EDS. H. P.

* * * * * The indifferent disposition manifested by physicians toward most medical periodicals is not wholly without cause. The daily necessity of the practitioner *is accurately verified therapeutic knowledge.* The "busy doctor" has no use for any other literature denominated medical. He will waste little time and less money on it; it goes to the waste basket if of any other character, often with a sigh of yearning for the fresh bread of therapeutics. He regards the subscribing and paying for a medical journal as a contract between himself and the publisher. He thinks he should have a journal devoted to the dissemination of professional knowledge. If a consistent homœopath, he cannot with interest consistently drum page after page laudatory of this "tonic" or that "sedative," etc. If he does desire such, he ought to experience little difficulty in making a selection, especially if he turns his optics toward Chicago. Homœopaths who are in earnest rightfully expect their journals to advocate practice founded on the principle which has given Homœopathy an abiding place in the hearts and homes of the most intelligent. The future interests of Homœopathy are unsafe in the hands of those who recommend these contraband drugs and compounds. If homœopaths *do* use them, they are inconsistent; if they do not, the advertisers of this class are most certainly poor financiers.

But if the medical journal does not come up to the standard of proper merit, is it altogether the fault of the publisher? Most certainly not. The medical publisher must necessarily make his publication financially successful. His columns must fill the exchequer. They are *offered* to the profession. But few of the many, ably competent, avail themselves of the privilege. How much it is to be regretted that a less number regard it a duty to contribute. This is *the* greatest and most potent reason that our medical journals are not more highly prized.

It is not the elaborately finished disquisition that makes an article the most acceptable to a practical reader. Let medical writers deliver fire at short range. Useful and practical information is at the command of every homœopathic practitioner. If he hides these under his bushel he is lacking in that spirit characteristic of a true physician. Such an one cannot say aught against the conduct of any medical journal.

The part that homœopathists shall have in the general prosperity will be measured by our present and future activity. *Doctor, you who read these lines, let the spirit of the immortal Hahnemann guide you; remember that the future of Homœopathy is not assured by the laurels won by its founders in the past.—The California Homœopath.*

